# Strategies for Improving Text Reading Ability Based on Human-Computer Interaction in Artificial Intelligence

**DOI:** 10.3389/fpsyg.2022.853066

**Published:** 2022-03-11

**Authors:** Guorong Shen

**Affiliations:** School of Foreign Languages, Henan University of Technology, Zhengzhou, China

**Keywords:** AI human-computer interaction, reading comprehension, neural network, attention mechanism, SQuAD dataset

## Abstract

In order to improve text reading ability, a human-computer interaction method based on artificial intelligence (AI) human-computer interaction is proposed. Firstly, the design of the AI human-computer interaction model is constructed, which includes the Stanford Question Answering Dataset (SQuAD) and the designed baseline model. There are three components: the coding layer is based on a cyclic neural network (recurrent neural network [RNN] encoder layer), which aims to encode the problem and text into a hidden state; the interaction layer is used to integrate problems and text representation; the output layer connects two independent soft Max layers after a fully connected layer, one is used to obtain the starting position of the answer in the text and the other is used to obtain the ending position. In the interaction layer of the model, this manuscript uses hierarchical attention and aggregation mechanism to improve text coding. The traditional model interaction layer has a simple structure, which leads to weak relevance between text and problems, and poor understanding ability of the model. Finally, the self-attention model is used to further enhance the feature representation of text. The experimental results show that the improved model in this manuscript is compared with the public AI human-computer interaction reading comprehension model. According to the data in the table, the accuracy of the model in this manuscript is better than that of the baseline model, in which the exact match (EM) value is increased by 1.4% and the F1 value is increased by 2.7%. However, compared with improvement point 2, the EM and F1 values of the model have decreased by 0.7%. It shows that the output layer has a certain impact on the effect of the model, and the improvement and optimization of the output layer can also improve the performance of the model. It is proved that AI human-computer interaction can effectively improve text reading ability.

## Introduction

Artificial intelligence (AI) has originated around 1950 and has gone through more than half a century. During this period, it experienced two declines and rose for the third time in recent years. As a new engine of the wave of global scientific change in the next stage, it is expected to lead the fourth industrial revolution and apply AI scientific research to various industries, which will bring broad development prospects. In recent years, China’s graded reading software has also begun to develop Chinese graded reading evaluation standards. Developers link evaluation standards with massive learning resources through AI technology to form a knowledge map. Therefore, students can be evaluated, intelligently graded, and predicted. The multi-terminal interactive design of excellent graded reading software provides convenience for the selection of excellent talents (Li et al., 2020). Students complete learning tasks through the student side, such as homework assigned by teachers, independent variant exercises, and a systematic review of wrong problem books. Through the parent side, parents can synchronously understand the learning track, data statistics, children’s wishes, strategy settings, etc. Teachers release learning tasks through the teacher side, track students’ learning progress, see each student’s quantifiable reading ability, and obtain visual data of class reading. The school can obtain the reading data report of the whole school through the headmaster, track the dynamic development of the school or regional reading level, and master the reading status of different grades and classes in real time, to facilitate macro-control and data analysis (Comunian, 2015). For example, check the poetry recitation ranking and student word breakthrough game ranking of all classes in the school, and select the list with the highest accuracy and the fastest speed. The school can also use this to select excellent talents. AI leads the future, which is also the general trend of future education development. Chinese teaching in primary schools is not only facing the challenge of conforming to the trend but also ushering in historical opportunities and a broader platform. Yu, an ancient educator, positioned teachers as “preaching, teaching and dispelling doubts.” The application of graded reading software in primary school Chinese teaching can replace some functions of teachers’ “teaching and dispelling doubts,” help to share some heavy and trivial work and reduce teachers’ teaching pressure. So that teachers can concentrate on more valuable work, that is, preaching and cultivate people with ideas, knowledge, wisdom, temperature, and soul (Martínez-Álvarez et al., 2015).

## Literature Review

Ungureanu first proposed the match LSTM and answer pointer model. Its core idea is to build a text representation based on question awareness through match LSTM, and then, predict the starting position of answer boundary through pointer network (Ungureanu et al., 2020). Kraska-Szlenk proposed a bidirectional attention flow (BiDAF) model. The model proposes a two-way attention mechanism, which hierarchically obtains text representation information with different granularity in multiple stages, and obtains document representation based on problem perception through a two-way attention flow mechanism (Kraska-Szlenk, 2018). Ulaiwy and Alarnoosy proposed the document reader model. The model does different processing for the word embedding layer of passage and question. In the word embedding layer of passage, traditional grammatical features, such as part of speech, word frequency, and named entity type, are added, while the question is simply processed, and good results are achieved with a relatively simple model architecture. At the same time, the author also constructs an open domain-based question answering system DrQA based on the document reader model (Ulaiwy and Alarnoosy, 2018). Xiong et al. (2016) proposed a cyclic neural network model. The greatest innovation of the recurrent neural network model is to apply the self-attention model to the reading comprehension task. Firstly, the model obtains the intermediate vector representation of the article with problem perception through the gated attention-based recurrent networks based on the attention mechanism and filters the impact of the problem on the article. Then, the self-attention mechanism is proposed to re-model the article. The self-attention mechanism can solve the problem that the recursive neural network is difficult to model the long-distance dependence in the text. The idea of the whole model can be formalized into the process of human reading comprehension: first, read the article with questions, preliminarily locate the article information related to the questions, then, browse an article again, screen out the real information that can answer the questions, and finally output the answers (Xiong et al., 2016). Renzulli proposed the FusionNet model. The model extends the existing attention methods from three aspects. Firstly, it proposes a new concept of “word history” to represent the attention information embedded from shallow words to the deep semantic level. Secondly, it identifies an attention scoring function that makes better use of the concept of “lexical history.” Finally, the model uses full awareness (full attention mechanism) at different levels, which not only uses the feature vectors of the current text and problems but also integrates the low-level feature vectors and high-level vectors through the aggregation mechanism as the input of the model output layer (Renzulli, 2015). Wu and Zhang proposed the SLQA model. The model integrates attention mechanism and fusion methods in each layer of the network. Firstly, the pre-trained language model Embeddings from Language Models (ELMo) is added to the word vector representation layer. Then, the combination of co-attention and fusion is used to represent articles and problems in the shallow layer, and the combination of self-attention and fusion is used in the deep layer. The main idea of the model is to refine layer by layer, decompose the complex task process of reading and understanding, and extract more useful feature information (Wu and Zhang, 2021). Santos and Canello proposed the Question Answering Network (QANet) model. The current end-to-end AI human-computer interaction reading comprehension models are based on recurrent neural networks (RNNs) with an attention mechanism. Although they have advantages in representing sequence information, the training of recurrent neural networks cannot be parallel, resulting in low training efficiency. As we all know, a convolutional neural network can extract local interactive information, and self-attention mechanism can obtain global interactive information (Santos and Canello, 2015). Rasinski et al. proposed a new question answering architecture. All coding layers in the model use convolutional neural network and self-attention mechanism to replace recursive neural network. Finally, compared with the model based on recurrent neural network, the effect is similar, and the training speed and reasoning speed are improved by about 10 times (Rasinski et al., 2016). Ma researchers proposed the pre-training language model Bidirectional Encoder Representation from Transformers (BERT). The pre-training model proposed by Google refreshed the best results of more than a dozen tasks in the field of natural language processing and improved the effect of the Stanford Question Answering Dataset (SQuAD) to exceed the human level. The release of pre-training model is a milestone in the field of natural language processing. Traditional word vectors, such as Word2Vec, are context independent. The word vector of each word will change with the task or training corpus. The word vector of the pre-training language model is context dependent, which can alleviate the dependence of specific tasks on the model structure (Ma, 2021).

**FIGURE 1:**
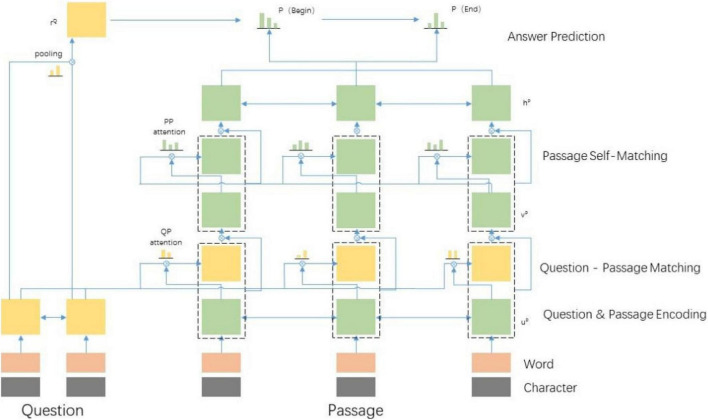
Artificial intelligence (AI)-human machine interaction.

Based on this, this manuscript proposes a human-computer interaction method based on AI human-computer interaction. Firstly, the design of the AI human-computer interaction model is constructed. As shown in Figure 1, it includes the SQuAD and the designed baseline model. The overall model has three components: RNN encoder layer based on cyclic neural network, which aims to encode problems and text into a hidden state. The interaction layer is used to integrate problems and text representations. The output layer connects two independent soft Max layers after a fully connected layer, one is used to obtain the starting position of the answer in the text and the other is used to obtain the ending position. In the interaction layer of the model, this manuscript uses hierarchical attention and aggregation mechanism to improve text coding. The traditional model interaction layer has a simple structure, which leads to weak relevance between text and problems, and poor understanding ability of the model. In order to extract more fine-grained text features, this manuscript first fuses the encoded results of the two two-way attention models and then, aggregates the low-level feature vectors into the current vector representation. Finally, the self-attention model is used to further enhance the feature representation of text.

## Artificial Intelligence Human-Computer Interaction Model Design

### Data Sets

The experiment uses the data set SQuAD, which contains about 100,000 triples in the form of text, questions, and answers, and SQuAD consists of 536 articles from Wikipedia. The articles are segmented manually. The generated natural segments are the text in the triple. An important feature of each data sample is that the answer always comes directly from the text, which means that the model does not need to generate the answer text, but only needs to intercept a text from the text corresponding to the question as the answer.

### Evaluation Index

The data set contains public training set and verification set. The verification set provides three answers to the questions of each sample, and each answer comes from different taggers. Therefore, the answers to the same question are not always completely consistent (Scammacca et al., 2015).

The performance of the model is measured by two indicators: F1 and exact match (EM) score.

EM (exact match) means exact match. It is used to detect whether the model output completely matches the real answer. There are only true and false results. For a question in a sample, if the model output answer is enough happiness and the real answer is have enough happiness, then the EM score of the model is 0.

Compared with EM, F1 is a relatively loose evaluation index, which is the harmonic average of precision and recall. In data science, precision measures how many of the answers given are real answers, and recall measures how many of the real answers are predicted. For the same example, if the output answer of the model is enough happiness and the real answer is have enough happiness, the model has 100% accuracy, because the real answer contains the prediction of the model, and the recall rate is 2/3, because only 2 of the 3 single word models in the real answer are given (Khalifian et al., 2015). As described above, according to the calculation formula of F1:


(1) $$F\,1=\frac{2\times P\times R}{P+R}$$

At this time, F1 score is 80%.

As mentioned earlier, since the validation set of the SQuAD has three human-provided answers to each question, the largest EM and F1 scores are selected from the three human-provided answers when evaluating the model performance. Using the above example, if one of the answers has indeed enough happiness, the model can obtain 100% EM and 100% F1 scores, which makes the evaluation more relaxed. Finally, average the whole data set to be evaluated to obtain the final EM and F1 scores (Arch and Stanhope, 2015).

### Baseline Model Design

For each sample of SQuAD, the pre-trained GloVe word vector is used to represent the text and problem words. The text is represented by a d-dimensional word vector sequence *x*_1_,…,*x*_*N*_ ∈ *R^d^* with length N, and the problem is represented by a d-dimensional word vector sequence *y*_1_,…,*y*_*M*_ ∈ *R^d^* with length M.

**FIGURE 2:**
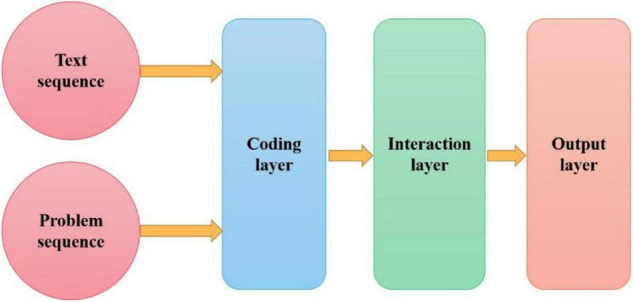
Schematic diagram of baseline model architecture.

The designed baseline model has three components: RNN encoder layer, which aims to encode problems and text into a hidden state. The interaction layer is used to integrate problems and text representations. The output layer connects two independent softmax layers after a full connection layer, one is used to obtain the starting position of the answer in the text and the other is used to obtain the ending position. Figure 2 shows the schematic diagram of the baseline model architecture.

#### Recurrent Neural Network-Based Coding Layer

The word vectors of the text and the question are sent to a single-layer bidirectional cyclic neural network Gated Recurrent Unit (GRU), as shown in the following formula:


(2) $$\left\{{\vec{c}}_{1},{\overset{\leftarrow }{c}}_{1},\ldots ,{\vec{c}}_{N},{\overset{\leftarrow }{c}}_{N}\right\}=biGRU\left(\left\{x_{1},\ldots x_{N}\right\}\right)$$

(3) $$\left\{{\vec{q}}_{1},{\overset{\leftarrow }{q}}_{1},\ldots ,{\vec{q}}_{N},{\overset{\leftarrow }{q}}_{N}\right\}=biGRU\left(\left\{y_{1},\ldots y_{M}\right\}\right)$$

Gated Recurrent Unit represents the dimension of hidden state. The bidirectional cyclic neural network GRU generates a series of text hidden states (${\vec{c}}_{i}\in R^{h}$ belongs to forward and ${\overset{\leftarrow }{c}}_{i}\in R^{h}$ belongs to backward) and a series of the problem hidden states (${\vec{q}}_{j}\in R^{h}$ belongs to forward and ${\overset{\leftarrow }{q}}_{j}\in R^{h}$ belongs to backward). The forward and backward hidden states are spliced to obtain the text hidden state *c*_*i*_ and problem hidden state *q*_*j*_ respectively:


(4) $$c_{i}=\left[{\vec{c}}_{i};{\overset{\leftarrow }{c}}_{i}\right]\in R^{2h}\;\forall i\in \left\{1,\ldots ,N\right\}$$

(5) $$q_{j}=\left[{\vec{q}}_{j};{\overset{\leftarrow }{q}}_{j}\right]\in R^{2h}\;\forall i\in \left\{1,\ldots ,M\right\}$$

#### Interaction Layer

The interaction layer is used to fuse text and problem information. It includes Context2Query from the text perspective and Query2Context from the text perspective. The former is used to obtain which words in which problems the text words focus on, and the latter is used to obtain which words in the text are more important for this problem (Choi et al., 2015).

Specifically, given the text hiding state *c*_1_,*c*_2_,…,*c*_*N*_ ∈ *R*^2*h*^ and problem hiding State *q*_1_,*q*_2_,…,*q*_*M*_ ∈ *R*^2*h*^ obtained from the coding layer, a similarity matrix *S* ∈ *R*^*N*×*M*^ is constructed, and each element *S*_*ij*_ ∈ *R* is obtained by the following formula:


(6) $$S_{i\,j}=w^{T}\,\left[c_{i};q_{j};c_{i}\times q_{j}\right]$$

Where *w* ∈ *R*^6*h*^ is a weight vector, × represents the element level product of two vectors. This similarity matrix s is used to create text to question interactions and problem to text interactions. Text to question interaction focuses on the question word most related to each text word (Jaquette and Curs, 2015). The calculation is as follows:


(7) $$a^{i}=s\,o\,f\,t\,\max\left(S_{i:}\right)\in R^{M}$$

(8) $$a_{i}=\sum_{j=1}^{M}a_{j}^{i}\,q_{j}\in R^{2\,h}\,\,\forall i\in \left\{1,\ldots ,N\right\}$$

The interactive calculation from question to text is as follows:


(9) $$\beta =s\,o\,f\,t\,\max\left(\underset{j}{\max S_{i\,j}}\right)\in R^{N}$$

(10) $$c^{\prime }=\sum_{i=1}^{N}\beta_{i}\,c_{i}\in R^{2\,h}$$

Representation of the last interaction:


(11) $$b_{i}=\left[c_{i};a_{i};c_{i}\times a_{i};c_{i}\times c^{\prime }\right]\in R^{8\,h}\,\begin{array}{cc} & \forall i\in \left\{1,\ldots ,N\right\} \end{array}$$

Enter *b*_*i*_ into a two-way GRU to get $b_{i}^{\prime }\in R^{2\,h},\forall i\in \left\{1,\ldots ,N\right\}$, which contains the interactive information of the corresponding word of the text about the whole text and the problem.

#### Output Layer

Each union represents $b_{i}^{{}^{\prime }}$ through a full connection layer and uses *ReLU* to activate the function:


(12) $$b_{i}^{\prime \prime }=R\,e\,L\,U\,\left(W_{F\,C}\,b_{i}^{\prime }+v_{F\,C}\right)\in R^{h}\,\begin{array}{cc} & \forall i\in \left\{1,\ldots ,N\right\} \end{array}$$

*W*_*FC*_ ∈ *R*^*h*×2*h*^ is the weight of the full connection layer and *v*_*FC*_ ∈ *R^h^* is the offset of the full connection layer. Pass all $b_{i}^{{}^{\prime \prime }}$ through a softmax layer to obtain the probability distribution of the starting position of an answer:


(13) $$s\,c\,o\,r\,e\,s_{i}^{s\,t\,a\,r\,t}=w_{s\,t\,a\,r\,t}^{T}\,b_{i}^{\prime \prime }+u_{s\,t\,a\,r\,t}\in R\,\begin{array}{cc} & \forall i\in \left[1,\ldots ,N\right] \end{array}$$

(14) $$p^{s\,t\,a\,r\,t}=s\,o\,f\,t\,\max\left(s\,c\,o\,r\,e\,s^{s\,t\,a\,r\,t}\right)\in R^{N}$$

Where *w*_*start*_ ∈ *R^h^* is a weight vector and *u*_*start*_ ∈ *R* is an offset term. Through the same softmax layer, using different parameters *w*_*end*_ and *u*_*end*_, the probability distribution of the end position of an answer is obtained.

#### Loss Function

For the real answer, the starting position in the text is *i*_*start*_ ∈ [1,…,*N*] and the ending position is *i*_*end*_ ∈ [1,…,*N*]. In the output layer, the probability distributions of the start position and the end position at n positions of the text can be obtained respectively. For a data sample, the cross-entropy loss is used to calculate the loss:


(15) $$l\,o\,s\,s=-\log p^{s\,t\,a\,r\,t}\,\left(i_{s\,t\,a\,r\,t}\right)-\log p^{e\,n\,d}\,\left(i_{e\,n\,d}\right)$$

In the training phase, the average loss is calculated on a batch of data (minibatch).

#### Forecast

In the prediction stage, given the text and problem of a sample, the output layer of the model can obtain the probability distributions of the start position and the end position at N positions of the text respectively.

First, calculate the indexes *idx^start^* and *idx^end^* of the start and end positions of the answer in the text as follows:


(16) $$i\,d\,x^{s\,t\,a\,r\,t}=\arg\max_{i=1}^{N}p_{i}^{s\,t\,a\,r\,t}$$

(17) $$i\,d\,x^{e\,n\,d}=\arg\max_{i=1}^{N}p_{i}^{e\,n\,d}$$

As shown above, using two independent softmax layers to predict *idx^start^* and *idx^end^* respectively, the *idx^start^* given by the model may be greater than *idx^end^*, to output a null answer.

Secondly, as a comparison, the length distribution of answers in the training data set is analyzed, and a sliding window with a unified length of W is set. In the prediction stage, given the text and question of a sample, take the first word in the sliding window as the starting position i. Set the other words in the sliding window as the termination position j and the computer probability $p_{i}^{s\,t\,a\,r\,t}\,p_{j}^{e\,n\,d}$ in turn and traverse the whole text in the sliding window so that the maximum i and j of $p_{i}^{s\,t\,a\,r\,t}\,p_{j}^{e\,n\,d}$ are *idx^start^* and *idx^end^*, respectively.

### Improved Model Design

The text is represented by a d-dimensional word vector sequence *x*_1_,…,*x*_*N*_ ∈ *R^d^* with length N, and the problem is represented by a d-dimensional word vector sequence *y*_1_,…,*y*_*M*_ ∈ *R^d^* with length M. therefore, the text X and problem y of a given sample can be represented as matrices of size *R*^*N*×*d*^ and *R*^*M*×*d*^ (Ribeiro et al., 2016).

**FIGURE 3:**
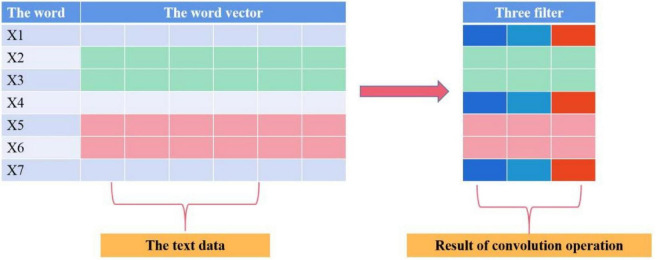
Schematic diagram of the convolution operation.

The length of the filter in the convolutional neural network (CNN) coding layer is equal to the word vector dimension d, and the width is set to k to cover k adjacent words, as shown in Figure 3.

The CNN coding layer is connected in series with K convolution layers, excluding the pooling layer. Each convolution layer has l filter. Based on the convolution operation of the previous layer, the width of the filter is continuously increased to obtain a wider range of receptive fields in the word dimension and extract a wider range of semantic information. Finally, the operation results of each convolution layer are spliced in the vector representation dimension of the word. The output dimension of text X through the CNN coding layer is *R*^*N*×(*l*×*K*)^, and the output dimension of problem y through the CNN coding layer is *R*^*M*×(*l*×*K*)^.

## Results and Analysis

At present, most AI human-computer interactive reading comprehension data sets are tested online. Therefore, developers cannot get the test set data. This makes it impossible for developers to frequently adjust model parameters and evaluate them on test sets. On the other hand, we observe that the models submitted by many authors have the same effect on the verification set and the test set, which shows that the samples of the verification set and the test set obey the same distribution. Therefore, it is a reasonable way to compare the performance of the model implemented in this manuscript by comparing the evaluation scores on the verification set (Stürmer et al., 2015).

The language model used in this manuscript is BERT, which is better than ELMo and OpenAI human-computer interaction generative pre-training model (GPT). The BERT model architecture uses a two-way multi-layer transformer encoder. The model training is different from the traditional language model to predict the next word as the target task. BERT proposes two new tasks. The first task is to hide 15% of words randomly in the output sequence and predict the hidden words, so that the model can predict the hidden words from any direction. Another task is to enable the model to learn the relationship between sentences and predict the next sentence. Bert can not only be directly applied to downstream tasks in the way of fine-tuning but also can apply the word vector obtained from model pre-training to model word vector initialization in the way of the feature. This manuscript adopts the second method, which uses BERT to extract fixed word vectors, which are obtained by the context representation of fixed length generated by the hidden layer of the pre-trained language model (Yang et al., 2015).

Firstly, the baseline model is reproduced, and the performance of the model is tested on the validation set. The experimental results are shown in Table 1.

**TABLE 1:** Baseline model evaluation results.

Method	EM	F1
Original paper effect	66.8%	80.6%
This paper realizes the effect	72.8%	81.5%

From the experimental results, the reproduced results in this manuscript are equivalent to the real results in the manuscript, and the score of F1 value is higher than 0.9% in the manuscript. Because the parameters of the model in the implementation process are not exactly the same as those in the original manuscript, there are also differences in the selection of attention function. Therefore, there are slight changes in the final results, but the overall model effect of the original manuscript is realized (Gaba et al., 2015).

In order to verify the effect of word vectors obtained by BERT on reading comprehension tasks, this manuscript makes a comparative experiment on the effect of BERT. The experimental results of the improved method of BERT based on the cyclic neural network model are shown in Table 2.

**TABLE 2:** Comparative experimental results based on Bidirectional Encoder Representation from Transformers (BERT).

Method	EM	F1
Original effect	69.3%	80.6%
Reproduction effect	72.4%	83.5%
Improved results based on BERT	72.6%	85.7%

It can be seen from the data in the table that the exact matching value of the cyclic neural network model added with BERT in the verification set has increased by 0.2%, and the F1 value has increased by 2.2%. It can be seen that the word vector generated by the language model trained by unsupervised learning has rich semantics. This context-sensitive word vector is very helpful to improve the effect of natural language understanding task. The word vector obtained by BERT has rich context and contains more lexical and syntactic information than the traditional word vector. In this manuscript, BERT is applied to the cyclic neural network model to verify the role of the original BERT (Sarah et al., 2015).

Then, the attention vector with problem influence based on word level is added to the representation of the text vector, and a three-layer bidirectional gated recurrent unit (BiGRU) network is used to encode the input vector in the coding layer. The network shares parameters between the whole text and the problem. FastText word vector has made three improvements on the basis of word2vec, so that the word vector contains more feature information, which is very helpful to eliminate ambiguity. This manuscript uses a FastText word vector with richer semantics instead of GloVe to calculate the word-level attention. The experimental results show that the FastText word vector is helpful to improve the experimental effect (Srivastava and Wang, 2015). It can be seen that the F1 value of the model is increased by 1.6% compared with the baseline model, which shows that the optimization and improvement of the word vector representation layer of the model can improve the quality of word vector representation, and then, improve the accuracy of the whole model. The experimental results are shown in Table 3.

**TABLE 3:** Comparison of experimental results based on word vector and context coding.

Method	EM	F1
Original effect	72.6%	80.7%
Reproducing results	71.6%	81.9%
Coding results based on word vector and context	73.6%	83.7%

### Statistical Analysis

**FIGURE 4:**
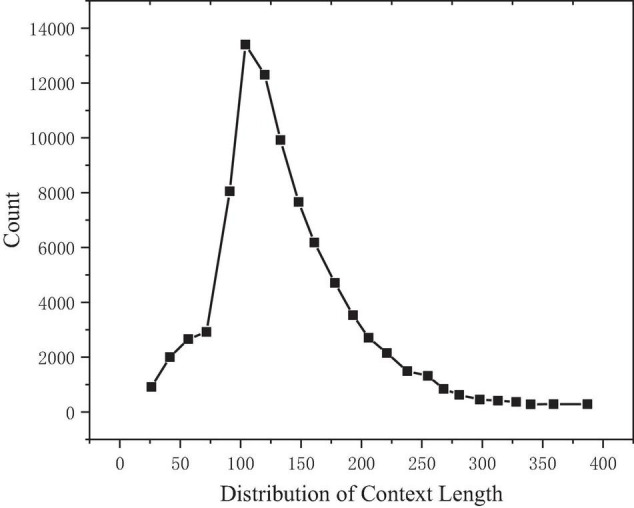
Text length distribution.

Make statistical analysis on the length of texts, questions, and answers of all samples in the training set. Taking the text length as the abscissa and the text count as the ordinate, it can be observed that the text length presents a right skew distribution, as shown in Figure 4. After calculation, the median length is 127, the average is 137, and the maximum value is 766, while 98.35% of the text length is concentrated in the range of 0–300.

**FIGURE 5:**
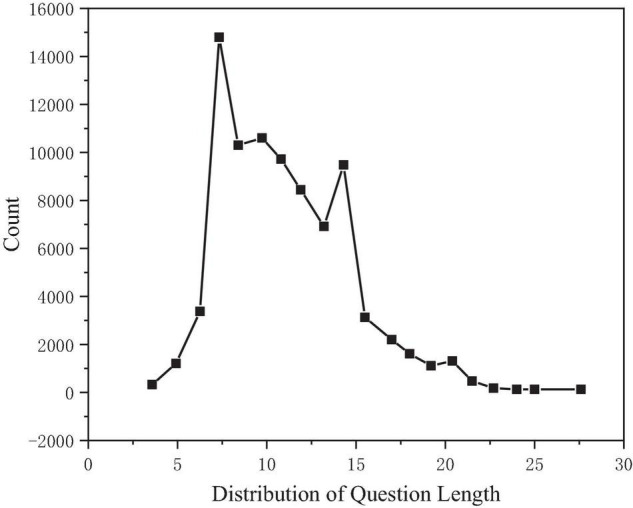
Problem length distribution.

Taking the problem length as the abscissa and the problem count as the ordinate, the distribution of the problem length can be observed, as shown in Figure 5. After calculation, the median length is 11, the average is 11, and the maximum value is 60, while 99.92% of the problem length is concentrated in the range of 0–30.

**FIGURE 6:**
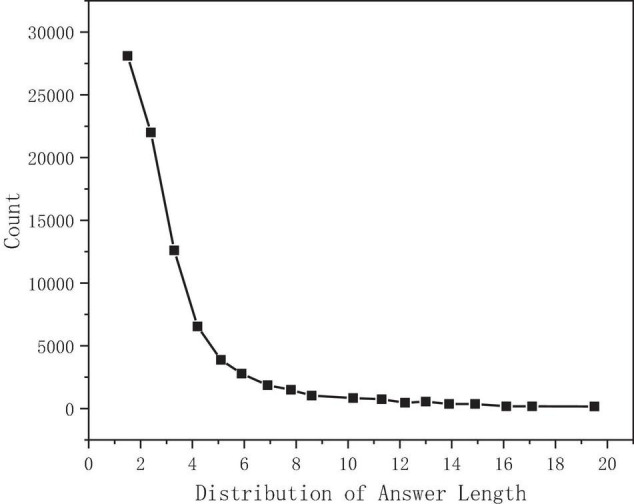
Answer length distribution.

Take the answer length as the abscissa and the answer count as the ordinate. The statistical distribution is shown in Figure 6. After calculation, the median length is 2, the average is 3, and the maximum value is 46, while 97.5% of the answer lengths are concentrated in the range of 0–15 (Dubreucq et al., 2016).

### Model Superparameters

According to the statistical analysis, 300 can cover 98.35% of the text length, the text length n is set to 300, 30 can cover 99.92% of the question length, the question length m is set to 30, 15 can cover 97.5% of the answer length, and the sliding window length W is set to 15. See Table 4 for details.

**TABLE 4:** Baseline model parameters.

Super parameter	Numerical value
Random inactivation	0.15
Text length N	300
Problem length M	30
Word vector dimension d	100
GRU hidden state dimension H (RNN coding layer)	150
GRU hidden state dimension H (interaction layer)	150
Sliding window length w	15

The CNN coding layer of the improved model is stacked with six convolution layers, and a larger filter is used for convolution operation on the convolution output of the previous layer. The super parameters are shown in Table 5.

**TABLE 5:** Super parameters of the improved model.

Super parameter	Numerical value
Convolution layer number K	6
Number of convolution layer filters l	50
Convolution layer filter width k	2, 3, 4, 5, 6, 7

### Model Training

The training batch size is set to 32, and the maximum gradient norm is set to 5. If it is greater than 5, gradient truncation is performed. Adam algorithm is used for training, and the initial learning rate is 0.001. The total learning rate after the 4,500th iteration of the baseline model is reduced to 0.0005, and the total learning rate after the 6,000th iteration is reduced to 0.0002. Based on the improved model of the CNN coding layer, the total learning rate after 2,500 iterations is reduced to 0.0008, the total learning rate after 11,000 iterations is reduced to 0.0005, the total learning rate after 16,000 iterations is reduced to 0.0002, and the total learning rate after 18,500 iterations is reduced to 0.0001 (Weiss et al., 2015).

#### Sliding Window

Firstly, verify the effect of the output layer on the baseline model on predicting the answer based on the sliding window and calculate the EM and F1 scores on the verification set respectively. The results are shown in Table 6.

**TABLE 6:** Effect verification of output layer based on sliding window prediction method.

Prediction mode	F1	EM
Not based on sliding window	68.7	58.0
Based on sliding window	70.7	59.7

#### Convolutional Neural Network Coding Layer

The baseline model uses RNN coding layer and the improved model uses the CNN coding layer. EM and F1 scores are calculated on the verification set respectively, as shown in Table 7.

**TABLE 7:** Comparison of exact match (EM) and F1 scores of baseline model and improved model.

Coding layer	F1	EM
RNN	70.7	59.7
CNN	66.9	54.9

**FIGURE 7:**
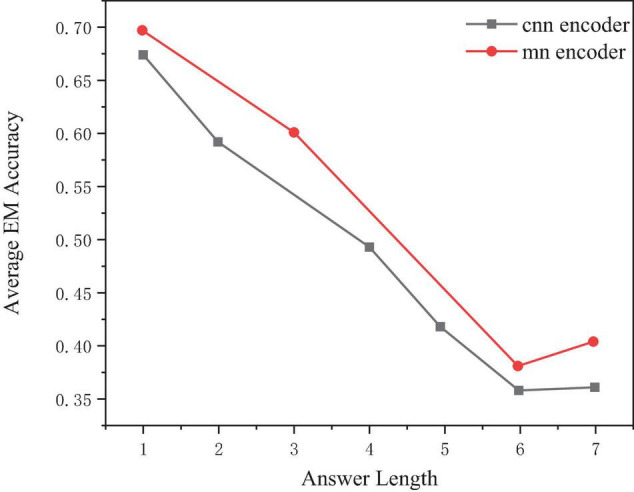
Comparison of exact match (EM) scores of two models on different length answers.

For different answer lengths, select 1–7 as an example, and the performance of the model will decrease with the increase of the real answer length, as shown in Figure 7.

Based on the improved scheme based on word vector representation and context coding, the model is further improved by combining the two-way attention and aggregation mechanism. In order to ensure the comparability of the model, this manuscript does not change the output layer structure of the model and still retains the same output layer structure as experiment 2. Through comparative analysis, the improved effect of the algorithm on attention and aggregation mechanism is verified. The experimental data are shown in Table 8.

**TABLE 8:** Comparison of experimental results based on attention and aggregation mechanism.

Method	EM	F1
Original effect	72%	79.9%
Reproduction effect	70.9%	80.6%
Results based on word vector and context coding	71.9%	83.7%
Results based on attention and aggregation mechanism	76.7%	89.8%

The experimental results in Table 8 show that after adding the hierarchical attention and aggregation mechanism, the effect of F1 value is improved by 11.8% based on the experiment of improved algorithm based on word vector and context and 7.9% higher than that of the original text. The method based on attention and aggregation mechanism can further improve the model’s ability to understand the text. Through attention and aggregation, the model can understand the text at different feature levels. However, the model has not significantly improved the evaluation index of accurate matching, which shows that there is still a certain deviation in the accurate positioning of multiple candidate answers in the text. The advantage of the attention mechanism is that it can locate the text position with the strongest relevance to the question, but the selection of multiple candidate answers depends on the differences of lexical features of the words themselves. The model needs to add more external knowledge to the representation layer to enhance the semantic expression of words. The answer area obtained by the attention mechanism is often accurate. However, to get a more accurate answer, we need more information about the word itself. Finally, combined with the improved methods of word vector representation layer and model interaction layer, this manuscript proposes an improved AI human-computer interactive reading comprehension model architecture. Moreover, further optimizes the reading comprehension model in this manuscript. This manuscript makes some changes to the output layer of the model based on the baseline model. There are two mainstream methods in the output layer of fragment extraction reading comprehension task. One is to predict the index of the beginning and end position of the answer through the pointer network or combined with the idea of dynamic programming, and the other is to use the two classification methods for prediction. This manuscript balances the advantages and disadvantages of the two methods and uses binary classification to replace the baseline model and uses pointer network to predict the answer. This prediction method may make the model lose certain accuracy, but the efficiency of model training and prediction will be improved a lot (Holmes et al., 2016).

After 45k iteration, the model tends to peak, and the model remains stable in the subsequent 15K iteration. In this manuscript, the experimental parameter values are updated every 1,000 steps, and the whole experimental training process is visualized by tensorboard. It shows that the output layer has a certain impact on the effect of the model, and the improvement and optimization of the output layer can also improve the performance of the model. In addition, this manuscript sacrifices a lot of time in exchange for the accuracy of the model when the experimental hardware allows, which is not desirable in practical engineering. Therefore, how to balance the relationship between model accuracy and training efficiency is a direction of follow-up improvement. In comparison with other public models, it can be seen that the performance of the AI human-computer interactive reading comprehension model implemented in this manuscript is higher than some classical models.

Finally, the comparative analysis shows that the word vectors extracted by language model pre-training can improve the effect of the model, which shows that the word vectors generated by language model pre-training have all rich semantics. At the same time, this manuscript uses the attention mechanism at different levels of the text. This manuscript uses word-level attention in the presentation layer and two-way attention and self-attention models in the model interaction layer. This multi-level attention mechanism can improve the semantic understanding ability of the model. Through experimental analysis, the improved methods of presentation layer and interaction layer can improve the effect of the model to varying degrees. Moreover, the accuracy of the improved AI human-computer interactive reading comprehension model is also better than the traditional model.

## Conclusion

Artificial intelligence human-computer interactive reading comprehension tasks have various forms. The SQuAD limits the answer to a fragment of the text. The existing models encode the text and questions based on a cyclic neural network to fuse the semantic information of the context. The main purpose of this manuscript is to try the coding layer based on multi-layer convolution operation. Through experimental analysis, combined with the improved model based on multi-layer convolution operation coding layer, the performance of the given evaluation index is slightly lower than the baseline model, but it is superior in the number of parameters and iteration speed, and the model design is reasonable. For the coding layer based on multi-layer convolution operation, the super parameter setting, such as the number of convolution layers, the number of filters, and the structure design, needs to be further explored. The SQuAD is a classic data set. The reading comprehension form of fragment extraction is relatively simple, while the application of AI human-computer interactive reading comprehension in the search engine, intelligent customer service, and other fields is more generative. Therefore, in the real world, AI human-computer interaction will face more complex and changeable data forms and a larger amount of data. The model needs a more detailed design and takes into account computational efficiency. In addition, human beings solve reading comprehension problems through reasoning, judgment, and induction and can express the thinking process. It is worth further expanding the traditional methods, such as visualization, to improve the interpretability of the model. Finally, with the rise of reinforcement learning, it also has some applications in the field of AI human-computer interactive reading comprehension. How to better combine it with practice and implement it to the application level is worth further exploration.

## Data Availability Statement

The original contributions presented in the study are included in the article/supplementary material, further inquiries can be directed to the corresponding author.

## Author Contributions

GS: editing, writing, and data analysis.

## Conflict of Interest

The author declares that the research was conducted in the absence of any commercial or financial relationships that could be construed as a potential conflict of interest.

## Publisher’s Note

All claims expressed in this article are solely those of the authors and do not necessarily represent those of their affiliated organizations, or those of the publisher, the editors and the reviewers. Any product that may be evaluated in this article, or claim that may be made by its manufacturer, is not guaranteed or endorsed by the publisher.
